# Oculodentodigital Dysplasia: A Case Report and Major Review of the Eye and Ocular Adnexa Features of 295 Reported Cases

**DOI:** 10.1155/2020/6535974

**Published:** 2020-04-04

**Authors:** Virang Kumar, Natario L. Couser, Arti Pandya

**Affiliations:** ^1^Virginia Commonwealth University School of Medicine, Richmond, VA, USA; ^2^Department of Ophthalmology, Virginia Commonwealth University School of Medicine, Richmond, VA, USA; ^3^Department of Human and Molecular Genetics, Virginia Commonwealth University School of Medicine, Richmond, VA, USA; ^4^Department of Pediatrics, Virginia Commonwealth University School of Medicine, Richmond, VA, USA; ^5^Department of Pediatrics, Division of Genetics and Metabolism, School of Medicine, University of North Carolina at Chapel Hill, Chapel Hill, NC, USA

## Abstract

Oculodentodigital dysplasia (ODDD) is a rare genetic disorder associated with a characteristic craniofacial profile with variable dental, limb, eye, and ocular adnexa abnormalities. We performed an extensive literature review to highlight key eye features in patients with ODDD and report a new case of a female patient with a heterozygous missense *GJA1* mutation (c.65G>A, p.G22E) and clinical features consistent with the condition. Our patient presented with multiple congenital anomalies including syndactyly, microphthalmia, microcornea, retrognathia, and a small nose with hypoplastic alae and prominent columella; in addition, an omphalocele defect was present, which has not been reported in previous cases. A systematic review of the published cases to date revealed 91 literature reports of 295 individuals with ODDD. There were 73 different *GJA1* mutations associated with these cases, of which the most common were the following missense mutations: c.605G>A (p.R202H) (11%), c.389T>C (p.I130T) (10%), and c.119C>T (p.A40V) (10%). Mutations most commonly affect the extracellular-1 and cytoplasmic-1 domains of connexin-43 (gene product of *GJA1*), predominately manifesting in microphthalmia and microcornea. The syndrome appears with an approximately equal sex ratio. The most common eye features reported among all mutations were microcornea, microphthalmia, short palpebral fissures, and glaucoma.

## 1. Introduction

Oculodentodigital dysplasia (ODDD, OMIM #164200) is a rare disorder mainly characterized by abnormal craniofacial, dental, ocular, and digital development. The autosomal dominant form has been the most frequently reported inheritance pattern, although a few cases of autosomal recessive inheritance have been described [1–3]. Craniofacial abnormalities may include microcephaly, prominent columella, and underdeveloped nasal alae [2–4]. Dental abnormalities, such as hypoplastic enamel, small teeth, and premature loss of teeth, are often present [2–4]. Digit abnormalities may include syndactyly, camptodactyly, and midphalangeal hypoplasia [2–4]. Ophthalmic manifestations are common, such as microcornea and microphthalmia, and may involve a wide spectrum of eye and ocular adnexa structures, although previous analyses of prior cases show that full ocular physical exams were not performed on all patients [3, 5].

The gap junction protein alpha 1 (*GJA1*) gene codes for connexin-43, which is a protein that assists in the transmembrane transport of molecules through gap junctions, and mutations in the GJA1 may cause an alteration of the channel conduction properties [1–3, 6]. We report a case of an 8-month-old female patient with an identified *GJA1* mutation and common clinical features associated with ODDD. This patient had an omphalocele at birth, which has not been reported in previous cases. Her eye features included microphthalmia, microcornea, narrow palpebral fissures, blonde fundus, deep anterior chambers, hyperopia, and epiphora in both eyes secondary to bilateral nasolacrimal duct obstructions. We conducted an extensive literature review to summarize the eye features in patients with ODDD reported to date.

## 2. Case Report

The patient, an 8-month-old female, was born to a nonconsanguineous couple from a healthy 37-year-old mother of Native American descent and a healthy 30-year-old father of German and Irish descent. Family history is notable for an older sibling with cleft palate, paternal uncle with autism, paternal second cousin with congenital heart defect, and distant paternal great-great uncle with Down syndrome and webbed/fused 4^th^ and 5^th^ digits of one hand. A normal pregnancy was noted until the second trimester when an omphalocele was detected on ultrasound. A subsequent ultrasound revealed possible syndactyly of the hands. The patient was born at 39 weeks by vaginal delivery with induction. The birth weight was 3.552 kg (75^th^ percentile), birth length was 50 cm (68^th^ percentile), and birth head circumference was 34.5 cm (70^th^ percentile). Apgar scores were 9 at both one minute and five minutes.

**Figure 1:**
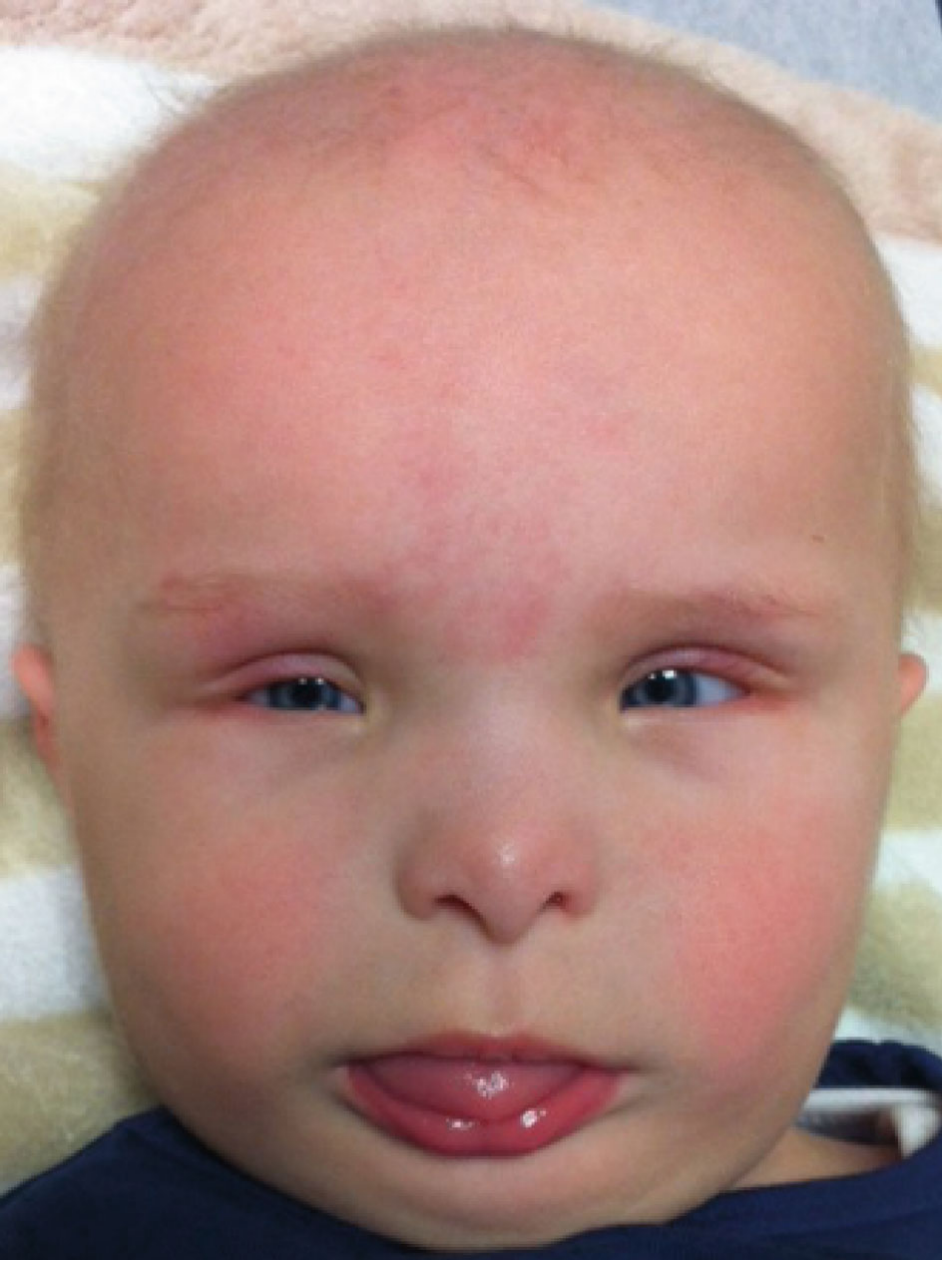
Facial photograph of a patient with oculodentodigital dysplasia; note the beaked nose with hypoplastic alae and prominent columella, microphthalmia, microcornea, small palpebral fissures, retrognathia.

**Figure 2:**
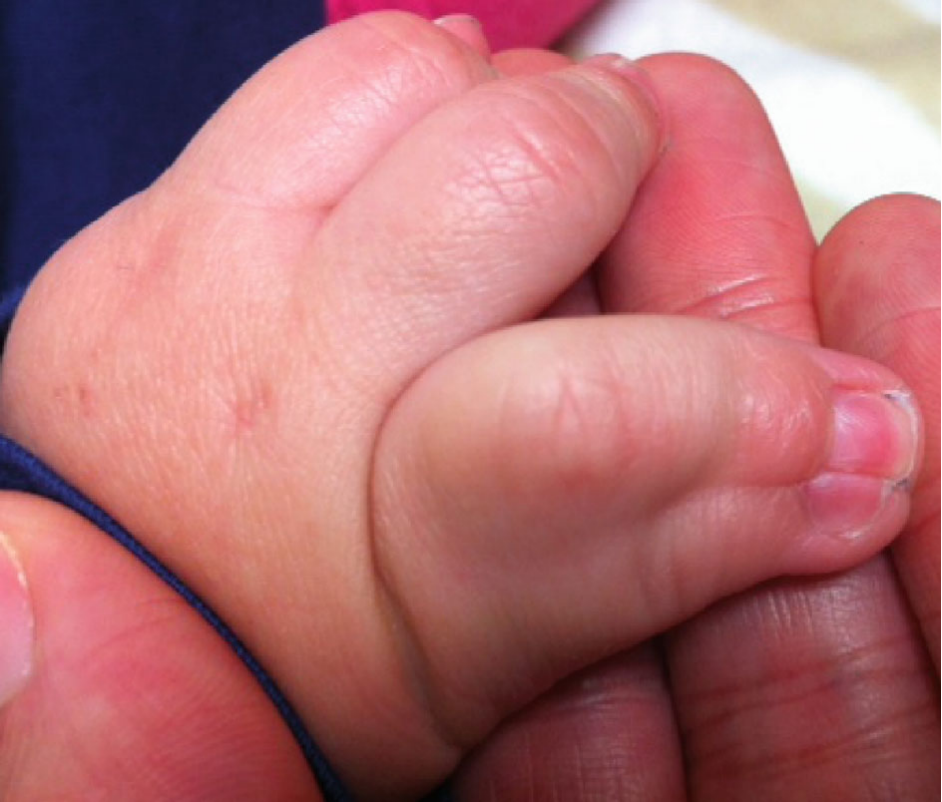
Complete syndactyly of the 4^th^ and 5^th^ digits of the right hand.

Multiple congenital anomalies noted at birth included an omphalocele that measured 4 cm at base and 3.5 cm across with intestines present in the sac, but no liver. The patient had a normocephalic head with sparse wispy hair, a small nose with hypoplastic alae, a prominent columella, small-appearing palpebral fissures, a small cornea, microphthalmia, a wide anterior fontanelle, and retrognathia (Figure 1). Syndactyly of digits 4 and 5 and webbing of digits 3 and 4 of the right (Figure 2) and left hands were present. Cardiac echocardiogram on the day of birth showed the presence of a mild patent ductus arteriosus, mild patent foramen ovale, and a normal aorta. Feeding difficulties were exacerbated by the presence of the omphalocele; surgical correction was performed on day 2 of life.

An ophthalmologic assessment at 4 months of age was notable for deep anterior chambers, bilateral nasolacrimal duct obstruction, microphthalmia, small 8 mm corneas, a blonde fundus, and moderate hyperopia in both eyes.

At her last examination at 8 months of age, the patient continues to have poor feeding with self-limiting volumes but has improved weight gain. The patient is at the 9^th^ percentile for weight and 12^th^ percentile for length. Cognitive and motor developments are delayed.

Sequencing of the *GJA1* gene (transcript number: NM_000165.3) from patient genomic DNA revealed a heterozygous missense mutation in the *GJA1* gene: c.65G>A (p.G22E). Deletion/duplication analysis of the *GJA1* gene using the aCGH test was negative.

## 3. Methods

We performed a systematic review of the literature to summarize the ocular findings in individuals with ODDD. A PubMed/Medline search of “oculodentodigital syndrome” led us to find a total of 177 articles. No articles were excluded based on the year published. We reviewed the references to identify other articles that did not appear in our original search. 91 articles describing patients with a description consistent with the clinical syndrome, either with or without molecular confirmation of *GJA1* pathogenic variants, were included. Within these selected articles, we identified 295 cases of ODDD with 73 different *GJA1* mutations, including those that exhibited features of ODDD in the absence of molecular confirmation. Such individuals were either clinically diagnosed or were relatives of individuals with molecularly confirmed *GJA1* pathogenic variants. Twelve reported that *GJA1* gene coding alterations were omitted due to insufficient clinical information and data reported and are listed in Table 1 [3, 6].

## 4. Discussion

Oculodentodigital dysplasia (ODDD) is a rare congenital disorder manifested with developmental anomalies of the eyes, face, dentition, heart, skeletal system, and digits. The syndrome appears to be more common in Caucasian populations with an equal sex ratio [3]. Heterozygous mutation of the *GJA1* gene located at chromosome 6q22.31 has been identified as the most common mutation resulting in ODDD [2, 3]. However, a compound heterozygous individual with missense mutations demonstrated mutations in the *GJA1* gene (p.V41L) and the *GJB2* gene (p.R127H), which encode for connexin-43 and connexin-26, respectively, and has been reported and classified as having overlapping features of Clouston syndrome and ODDD [3, 7].

In addition to the classic phenotypic features of the syndrome, a wide variety of additional physical manifestations have been observed. Ocular findings of microphthalmia and microcornea have been observed commonly in previous cases [2–4]. Craniofacial anomalies of microcephaly, poor hair growth, hypoplastic nasal alae, and prominent columella have been reported previously [2–4]. Bilateral syndactyly of the 4^th^ and 5^th^ digits is common [2, 3].

A systematic review of the published cases to date (ranging from 1963 to 2019) revealed 91 literature reports of 295 individuals with ODDD [1–91].  Table 2 [1–91] summarizes the sex distribution across all reviewed reports of ODDD. Patients with ODDD present with an approximately equal sex distribution (47% male and 53% female). Of the 295 individuals reported, 32 were clinically diagnosed with ODDD without molecular confirmation, 98 presented with features of ODDD and had a known relative with molecular confirmation of a *GJA1* pathogenic variant, and 165 individuals had a molecularly confirmed *GJA1* pathogenic variant.

There were 73 different *GJA1* mutations identified from the 165 individuals that had a molecularly confirmed *GJA1* pathogenic variant.  Table 3 [1–3, 5–71, 92] summarizes the number of patients with each mutation. Patients with confirmed pathogenic variants and their relatives with no molecular confirmation but with features of ODDD were grouped separately. These two groups comprised 263 of the patients included in this study.

The eye features of all 295 patients are summarized in Table 4 [1–91]. The most common ophthalmic manifestations reported were microcornea (*n* = 111), microphthalmia (*n* = 110), short palpebral fissures (*n* = 56), and glaucoma (*n* = 51, 4 closed-angle and 1 open-angle).

Twenty-three patients presented with refractive error, of which isolated myopia was the most frequently noted (*n* = 14), followed by isolated hyperopia (*n* = 6), anisometropia (*n* = 2), and astigmatism (*n* = 1). Forty patients presented with eye movement disorders, with strabismus (*n* = 27, 9 esotropic, 1 exotropic) being the most common, followed by nystagmus (*n* = 8), amblyopia (*n* = 3), Duane syndrome (*n* = 2), and Brown syndrome (*n* = 1). Note that 1 patient had both nystagmus and esotropia [71]. Other common findings included epicanthus (*n* = 36), hypotelorism (*n* = 24), hypertelorism (*n* = 22), madarosis (*n* = 19), cataracts (*n* = 17), persistent pupillary membranes (*n* = 13), shallow anterior chambers (*n* = 12), pale/atrophic irides (*n* = 11), telecanthus (*n* = 11), and uveitis (*n* = 10).

A variety of abnormal findings for the retina and optic disc were noted (*n* = 18), with dysplasia of the retina/fundus (*n* = 3) and pale/atrophic optic discs (*n* = 3) being the most common documented findings.

Of the individuals with molecularly confirmed mutations, the most common mutations present were c.605G>A (p.R202H) (11%; with 1 patient also having a c.717G>A synonymous mutation), c.389T>C (p.I130T) (10%), and c.119C>T (p.A40V) (10%).  Table 5 [2, 3, 12, 30, 40, 41, 66, 67, 92] summarizes the eye features present in the patients with these mutations.

Less common features of the phenotype observed in our presented case were also reported in other cases as well. These include nasolacrimal duct abnormalities (*n* = 2), pale/atrophic retina/fundus (*n* = 2), and deep anterior chambers (*n* = 2). Additionally, including this study, the three patients with the p.G22E mutation have the following findings: microphthalmia (*n* = 3), cataracts (*n* = 1), microcornea (*n* = 2), blonde fundus (*n* = 1), persistent pupillary membrane (*n* = 1), deep anterior chamber (*n* = 1), hyperopia (*n* = 1), strabismus (*n* = 2, 1 esotropic), amblyopia (*n* = 1), glaucoma (*n* = 1), short palpebral fissures (*n* = 1), nasolacrimal duct abnormalities (*n* = 1), and epicanthus (*n* = 1) [2, 3, 21, 22].

Some unique genotype-phenotype correlations were noted upon further analysis. Three patients presented with eccentric pupils, but only 2 of these patients were reported with an associated mutation. Both mutations (p.Q49dup and p.Q49P) seem to affect the same amino acid in connexin-43 [3, 61, 72]. Additionally, uveitis was reported in 10 patients, 9 of which were associated with similar mutations. Eight of these patients were within the same study and had the p.H194P mutation, another patient had no molecular confirmation of a *GJA1* mutation, and the other patient was reported with a missense mutation on exon 2 [4, 9, 10, 27, 28]. However, since the majority of these patients were reported within the same study, the apparent genotype-phenotype correlation of p.H194P and uveitis might be due to underreporting of uveitis from other sources with different pathogenic variants or may be due to other factors of the family not identified within the study.

Further analysis of the genotype-phenotype correlation was conducted by pairing the phenotypic manifestations of each mutation with the corresponding defects in the connexin-43 domains. The domains were defined by the amino acid ranges provided on UniProt (P17302–CXA1_HUMAN) [93].  Table 6 [1–3, 5–71, 92, 93] provides a summary of the phenotypes associated with mutations from each domain.

The domains most commonly affected by *GJA1* mutations are the extracellular-1 loop and the cytoplasmic-1 loop of connexin-43, accounting for 19 and 20 mutations, respectively. Disruptions in the extracellular-1 loop presented primarily as microphthalmia (*n* = 32) and microcornea (*n* = 30). A similar pattern can be seen in the cytoplasmic-1 loop, as the most common presentations were microphthalmia (*n* = 20) and microcornea (*n* = 18). Other clinical findings, however, may be able to distinguish mutations resulting from these domains. The next most common findings associated with mutations in the extracellular-1 loop were glaucoma (*n* = 15) and hypertelorism (*n* = 11), as opposed to short palpebral fissures (*n* = 14) and hypotelorism (*n* = 14) for the cytoplasmic-1 loop.

Mutations affecting the cytoplasmic N-terminus and the transmembrane-1 domain shared similar features to the ones in the extracellular-1 and cytoplasmic-1 domains, as microphthalmia and microcornea were the most common clinical findings. However, the mutations in the cytoplasmic N-terminus and transmembrane-1 domain presented with microcornea (*n* = 17 and *n* = 21, respectively) more frequently than microphthalmia (*n* = 5 and *n* = 14, respectively). The opposite pattern is true for the extracellular-1 and cytoplasmic-1 domains.

The mutations in the extracellular-2 loop demonstrate a different phenotypic pattern, as microphthalmia (*n* = 14) occurs the most frequently, while microcornea is less frequent (*n* = 4). Mutations in the transmembrane-2 domain also display a unique pattern, with hypertelorism (*n* = 5) being the most frequent clinical finding. Other domains listed in Table 6 also demonstrate some unique clinical patterns, but this may be due to variability from the small number of samples. The patterns mentioned previously, however, still provide insight into the role of different connexin-43 domains in providing phenotypic variability among patients with ODDD.

In conclusion, this report provides a comprehensive review of the eye and ocular adnexa abnormalities that are currently known to be associated with the ODDD phenotype. Limitations of this report include the possibility of an incomplete ophthalmologic evaluation and/or lack of reporting of eye features in all of the evaluated case reports or misdiagnosis in the individuals with the ODDD phenotype without molecular confirmation. As such, it is possible that the reported common eye features within this summary may be over or underrepresented. Ophthalmic manifestations are commonly associated within the phenotype, and a wide spectrum of eye and ocular adnexa structures may be affected. The rarity of this condition provides further incentive to further investigate the phenotype.

## Figures and Tables

**Table 1:** *GJA1* variants without clinical information.

Sources	*GJA1* variant		Cases
	Nucleotide	Protein	
Paznekas et al. [3]	c.7G>A	p.D3N	1
Paznekas et al. [3]	c.64G>A	p.G22R	1
Paznekas et al. [3]; Richardson et al. [6]	c.79T>C	p.S27P	1
Paznekas et al. [3]	c.163A>G	p.N55D	1
Paznekas et al. [3]	c.174A>C	p.Q58H	1
Paznekas et al. [3]	c.175C>G	p.P59A	1
Paznekas et al. [3]	c.221A>T	p.H74L	1
Paznekas et al. [3]	c.428G>A	p.G143D	1
Paznekas et al. [3]	c.430A>G	p.K144E	1
Paznekas et al. [3]	c.434T>G	p.V145G	1
Paznekas et al. [3]	c.442C>G	p.R148G	1
Paznekas et al. [3]	c.578C>T	p.P193L	1

**Table 2:** Summary of sex distribution.

	Males		Females		Total
Individuals with clinical diagnosis of ODDD (with no molecular confirmation)	14	45%	18	56%	32

Untested individuals with both ODDD phenotype and known relative with molecular confirmation	52	53%	46	47%	98

Individuals with a molecular confirmed *GJA1* pathogenic variant	72	44%	93	56%	165

Totals	138	47%	157	53%	295

**Table 3:** Reported *GJA1* mutations and sex distribution in ODDD.

Sources	Multiple mutations?	*GJA1* mutation			Individuals with a molecular confirmed *GJA1* pathogenic variant		Untested individuals with both ODDD phenotype and known relative with molecular confirmation		Total individuals with the ODDD phenotype				
		Nucleotide	Protein	Unspecified	Male	Female	Male	Female	Male		Female		Total
Cavusoglu et al. 2019	No	c.168_169insT	p.Q57SfsTer6	N/A	1	0	0	0	1	100%	0	0%	1
Aminabadi et al. 2009 & Aminabadi et al. 2010	No	N/A	N/A	Missense mutation exon 2 (unspecified)	1	0	2	1	3	75%	1	25%	4
Dwarakanathan et al. 2015 & Furuta et al. 2012	No	c.75G>T	p.W25C	N/A	1	1	0	0	1	50%	1	50%	2
Quick and Dobersen 2014; National Center for Biotechnology Information 2020	Yes	c.605G>A	p.R202H	N/A	1	0	0	0	1	100%	0	0%	1
		c.717G>A	p.R239R										
Paznekas et al. 2003 & Paznekas et al. 2009	No	c.605G>A	p.R202H	N/A	1	7	4	5	5	29%	12	71%	17
Jamsheer et al. 2010	Yes	c.301C>T	p.R101X	N/A	1	0	0	0	1	100%	0	0%	1
		c.6delT	p.G2fsX7										
Jamsheer et al. 2010	No	c.301C>T	p.R101X	N/A	0	1	0	0	0	0%	1	100%	1
Paznekas et al. 2009; Joss et al. 2008; & Richardson et al. 2006	No	c.97C>T	p.R33X^∗^	N/A	0	2	0	0	0	0%	2	100%	2
Paznekas et al. 2009; Richardson et al. 2004; Paznekas et al. 2003; & Gladwin et al. 1997	No	c.93T>C	p.I31M	N/A	0	0	4	4	4	50%	4	50%	8
Wang et al. 2019	No	c.91A>T	p.I311P	N/A	1	0	0	0	1	100%	0	0%	1
Paznekas et al. 2009 & van Steensel et al. 2005	No	c.780_781delTG	p.C260fsX306	N/A	1	2	0	0	1	33%	2	67%	3
Paznekas et al. 2009; Paznekas et al. 2003; & Gorlin et al. 1963	No	c.68A>C	p.K23T	N/A	1	0	0	0	1	100%	0	0%	1
Dwarakanathan et al. 2015; Paznekas et al. 2009; & Vreeburg et al. 2007	No	c.689_690delAT	p.Y230fsX236	N/A	0	3	1	0	1	25%	3	75%	4
This study; Gumus 2018; Paznekas et al. 2009; Paznekas et al. 2003; & Traboulsi and Parks 1990	No	c.65G>A	p.G22E	N/A	0	3	0	0	0	0%	3	100%	3
Wiest et al. 2006	No	c.659C>A	p.S220Y	N/A	0	1	0	0	0	0%	1	100%	1
Paznekas et al. 2009; Paznekas et al. 2003; & Norton et al. 1995	No	c.646G>T	p.V216L	N/A	1	0	4	1	5	83%	1	17%	6
Park et al. 2017; Paznekas et al. 2009; & Paznekas et al. 2003	No	c.61G>A	p.G21R	N/A	0	2	0	0	0	0%	2	100%	2
Brice et al. 2013	No	c.617A>G	p.K206R	N/A	1	2	1	1	2	40%	3	60%	5
Paznekas et al. 2009	No	c.602C>T	p.S201F	N/A	0	1	0	0	0	0%	1	100%	1
Paznekas et al. 2009 & de la Parra et al. 2007	No	c.5G>T	p.G2V	N/A	1	0	0	0	1	100%	0	0%	1
Vitiello et al. 2005 & Vingolo et al. 1994	No	c.581A>C	p.H194P^∗^	N/A	3	5	3	3	6	43%	8	57%	14
Paznekas et al. 2009; Paznekas et al. 2003; & Judisch et al. 1979	No	c.52T>C	p.S18P	N/A	0	0	1	3	1	25%	3	75%	4
Paznekas et al. 2009 & Paznekas et al. 2003	No	c.50A>C	p.Y17S	N/A	3	4	0	0	3	43%	4	57%	7
Paznekas et al. 2009 & Debeer et al. 2005	No	c.504_506delCTT	p.F169del	N/A	0	1	0	0	0	0%	1	100%	1
Wiest et al. 2006 & Thomsen et al. 1998	No	c.461C>A	p.T154N	N/A	0	2	0	1	0	0%	3	100%	3
Paznekas et al. 2009 & van Es et al. 2007	No	c.460A>G	p.T154A^∗^	N/A	0	2	0	0	0	0%	2	100%	2
Paznekas et al. 2009; Richardson et al. 2004; Paznekas et al. 2003; Gladwin et al. 1997; & Schrander-Stumpel et al. 1993	No	c.443G>A	p.R148Q	N/A	0	0	2	2	2	50%	2	50%	4
Taşdelen et al. 2018	No	c.442C>T	p.R148Ter	N/A	1	0	0	0	1	100%	0	0%	1
Paznekas et al. 2009; Debeer et al. 2005; & Spaepen et al. 1991	No	c.440Y>C	p.M147T	N/A	0	1	0	0	0	0%	1	100%	1
Paznekas et al. 2009; Richardson et al. 2004; & Brueton et al. 1990	No	c.427G>A	p.G143S	N/A	0	0	8	1	8	89%	1	11%	9
Orosz et al. 2018	No	c.413G>A	p.G138D	N/A	1	0	0	0	1	100%	0	0%	1
Paznekas et al. 2009; Paznekas et al. 2003; & Shapiro et al. 1997	No	c.412G>C	p.G138R	N/A	1	2	2	2	3	43%	4	57%	7
Kogame et al. 2014	No	c.412G>A	p.G138S	N/A	1	0	0	0	1	100%	0	0%	1
Paznekas et al. 2009; Richardson et al. 2004; Paznekas et al. 2003; & Gladwin et al. 1997	No	c.402G>T	p.K134N	N/A	0	0	0	2	0	0%	2	100%	2
Paznekas et al. 2009 & Paznekas et al. 2003	No	c.400A>G	p.K134E	N/A	0	1	0	0	0	0%	1	100%	1
Nishat et al. 2012; Paznekas et al. 2009; Paznekas et al. 2003; & Amador et al. 2008	No	c.389T>C	p.I130T	N/A	7	4	5	1	12	71%	5	29%	17
Paznekas et al. 2009; Musa et al. 2008; Wiest et al. 2006; & Loddenkemper et al. 2002	No	c.338T>C	p.L113P	N/A	2	2	1	0	3	60%	2	40%	5
Paznekas et al. 2009 & Debeer et al. 2005	No	c.330G>C	p.E110D	N/A	2	3	1	2	3	38%	5	63%	8
Paznekas et al. 2009 & Kelly et al. 2006	No	c.32T>C	p.L11P	N/A	0	1	0	0	0	0%	1	100%	1
Gabriel et al. 2011 & Jamsheer et al. 2009	No	c.31C>T	p.L11F	N/A	0	2	0	0	0	0%	2	100%	2
Porntaveetus et al. 2017	No	c.31C>A	p.L11I	N/A	1	0	0	0	1	100%	0	0%	1
Jamsheer et al. 2014	No	c.317T>G	p.L106R	N/A	2	0	0	0	2	100%	0	0%	2
Paznekas et al. 2009 & Nivelon-Chevallier et al. 1981	No	c.317T>C	p.L106P	N/A	1	0	0	0	1	100%	0	0%	1
Paznekas et al. 2009 & Paznekas et al. 2003	No	c.306G>C	p.K102N	N/A	1	2	0	0	1	33%	2	67%	3
Paznekas et al. 2009; Paznekas et al. 2003; & Wooldridge et al. 1977	No	c.293A>G	p.Y98C	N/A	1	3	1	1	2	33%	4	67%	6
Paznekas et al. 2009	No	c.287T>C	p.V96A	N/A	0	1	0	0	0	0%	1	100%	1
Wiest et al. 2006	No	c.287T>A	p.V96E	N/A	0	1	0	0	0	0%	1	100%	1
Paznekas et al. 2009 & Kjaer et al. 2004	No	c.286G>A	p.V96M	N/A	2	2	0	0	2	50%	2	50%	4
Paznekas et al. 2009 & Honkaniemi et al. 2005	No	c.284A>G	p.H95R	N/A	0	1	0	1	0	0%	2	100%	2
Paznekas et al. 2009; Paznekas et al. 2003; & Opjordsmoen and Nyberg-Hansen 1980	No	c.268C>G	p.L90V	N/A	4	0	3	2	7	78%	2	22%	9
Jamsheer et al. 2014	No	c.257C>A	p.S86Y	N/A	0	1	0	0	0	0%	1	100%	1
Pizzuti et al. 2004	No	c.227G>A	p.R76H	N/A	1	0	0	0	1	100%	0	0%	1
Izumi et al. 2013	No	c.226C>T	p.R76C	N/A	1	0	0	0	1	100%	0	0%	1
Paznekas et al. 2009; Paznekas et al. 2003; & Stanislaw et al. 1998	No	c.226C>A	p.R76S	N/A	0	2	0	2	0	0%	4	100%	4
Choi et al. 2018	No	c.221A>C	p.H74P^∗^	N/A	1	0	0	0	1	100%	0	0%	1
Paznekas et al. 2009; Richardson et al. 2004; Paznekas et al. 2003; & Gladwin et al. 1997	No	c.206C>A	p.S69Y	N/A	0	0	2	5	2	29%	5	71%	7
Paznekas et al. 2009 & Vasconcellos et al. 2005	No	c.176C>A	p.P59H	N/A	4	4	1	0	5	56%	4	44%	9
Paznekas et al. 2009	No	c.145_147dupCAG	p.Q49dup	N/A	0	1	0	0	0	0%	1	100%	1
Pazenkas et al. 2009; Paznekas et al. 2003; Weintraub et al. 1975; & Gellis and Feingold 1974	No	c.154_156dupTTT	p.F52dup	N/A	1	0	1	1	2	67%	1	33%	3
Hadjichristou et al. 2017 & Paznekas et al. 2009	No	c.146A>C	p.Q49P	N/A	1	1	0	0	1	50%	1	50%	2
Izumi et al. 2013	No	c.145C>G	p.Q49E	N/A	0	1	0	0	0	0%	1	100%	1
Paznekas et al. 2009 & Paznekas et al. 2003	No	c.145C>A	p.Q49K	N/A	3	2	0	0	3	60%	2	40%	5
Amano et al. 2012; Feller et al. 2008; Paznekas et al. 2009; & Itro et al. 2005	No	c.142G>A	p.E48K	N/A	3	0	0	0	3	100%	0	0%	3
Jamsheer et al. 2014	No	c.139G>C	p.D47H	N/A	0	3	0	0	0	0%	3	100%	3
Tumminelli et al. 2016	No	c.125G>C	p.E42Q	N/A	1	0	0	0	1	100%	0	0%	1
Gabriel et al. 2011	No	c.120delGGTTGAGTCAGC	p.V41_A44del	N/A	0	1	1	2	1	25%	3	75%	4
Paznekas et al. 2009 & Kellermayer et al. 2005	Yes (compound heterozygous with *GJB2* mutation)	c.121G>C	p.V41L	N/A	0	1	0	0	0	0%	1	100%	1
		N/A	p.R127H (*GJB2* mutation)										
Park et al. 2019; Hayashi et al. 2014; Paznekas et al. 2009; Debeer et al. 2005; & Paznekas et al. 2003	No	c.119C>T	p.A40V	N/A	6	4	4	3	10	59%	7	41%	17
Wittlieb-Weber et al. 2015	No	c. 175C>T	p.P59S	N/A	1	2	0	0	1	33%	2	67%	3
Attig et al. 2016	No	c.396_398delAAA	p.I132_K133delinsM	N/A	3	2	0	0	3	60%	2	40%	5
Paznekas et al. 2009	No	c.19T>G	p.L7V	N/A	1	0	0	0	1	100%	0	0%	1
Himi et al. 2009	No	c.13A>T	p.S5C	N/A	0	1	0	0	0	0%	1	100%	1
Pace et al. 2019	No	c.287T>G	p.V96G	N/A	0	1	0	0	0	0%	1	100%	1
	No	c.77T>C	p.L26P	N/A	0	1	0	0	0	0%	1	100%	1
Totals					72	93	52	46	124	47%	139	53%	263

^∗^Unknown which specific individuals tested.

**Table 4:** Eye and ocular adnexa features reported in ODDD.

Orbit	—	Microphthalmia (110/37%)	Hypotelorism (24/8%)	Hypertelorism (22/7%)	Short axial length (4/1%)						
Anterior segment	Anterior chamber	Shallow anterior chamber (12/4%)	Deep anterior chambers (2/<1%)								
	Cornea	Microcornea (111/38%)	Thick corneas (4/1%)	Corneal opacities (3/1%)	Corneal farinata (1/<1%)	Band keratopathy (1/<1%)	Corneal keratosis (1/<1%)	Abnormal Descemet's membrane (1/<1%)	Anteriorly deviated Schwalbe's line (1/<1%)		
	Sclera	Blue sclera (1/<1%)									
	Pupil	Persistent pupillary membranes (13/4%)	Eccentric pupils (3/1%)								
	Lens	Cataracts (17/6%)	Lens opacities (2/<1%)	White retrolental masses (1/<1%)							
	Uvea (iris, ciliary body)	Pale/atrophic irides (11/4%)	Uveitis (10/3%)	General iris abnormalities (7/2%)	Synechiae (4/1%)	Hypoplastic anterior iris stroma (3/1%)	Ciliary body cysts (2/<1%)	Flat iris (1/<1%)	Iridoschisis (1/<1%)	Inferior iris coloboma (1/<1%)	Dysplastic iris (1/<1%)

Posterior segment	Uvea (choroid)	Thick choroid (2/<1%)	Thin choroid (1/<1%)								
	Vitreous	Vitreous degeneration (1/<1%)	Vitreous membrane attachment to optic nerve and lens (1/<1%)	Persistent hyperplastic primary vitreous (1/<1%)							
	Retina/fundus	Dysplastic retina/fundus (3/1%)	Pale retina/fundus (2/<1%)	Thread-like retinal vasculature (2/<1%)	Dystrophic retinal epithelium (1/<1%)	Hypoplastic macula (1/<1%)	Absent fundal glow with B-scan ultrasound (1/<1%)				
	Optic disc	Pale/atrophic optic disc (3/1%)	Dysplastic optic disc (2/<1%)	Ellipsoid optic disc (1/<1%)	Optociliary vein presence (1/<1%)	Optic disc hypervascularity (1/<1%)					

Ocular adnexa	Eyelid	Short/narrow palpebral fissures (56/19%)	Epicanthus (36/12%)	Telecanthus (11/4%)	Ptosis (7/2%)	Blepharophimosis (1/<1%)	Entropion (1/<1%)	Ectropion (1/<1%)	Epiblepharon (1/<1%)	Mucosal hypertrophy (1/<1%)	
	Eyebrow/eyelash	Madarosis (19/6%)	Flared eyebrows (3/1%) (2 medially flared)	Synophyrs (1/<1%)							
	Nasolacrimal duct	Nasolacrimal duct abnormalities (2/<1%)	Hypolacrimation (1/<1%)								

Other	Refractive errors	Myopia (16/5%) (2 anisometropic)	Hyperopia (8/3%) (2 anisometropic)	Astigmatism (1/<1%)							
	Eye movement disorders	Strabismus (27/9%) (9 esotropic, 1 exotropic)	Nystagmus (8/3%)	Amblyopia (3/1%)	Duane syndrome (2/<1%)	Brown syndrome (1/<1%)					
	Additional eye disorders	Glaucoma (51/17%) (4 closed-angle, 1 open-angle)	Paracentral scotoma (1/<1%)								
	ERG/neurological	Abnormal ERG (2/<1%)	Delayed visual evoked responses (2/<1%)	Occipital subcortical white matter changes (1/<1%)							

**Table 5:** Common *GJA1* mutations with associated eye features.

Sources	Multiple mutations?	*GJA1* mutation		Individuals with *GJA1* mutation (confirmed and affected relatives)	Associated eye features
		Nucleotide	Protein	Total	
Quick and Dobersen 2014; National Center for Biotechnology Information 2020	Yes	c.605G>A	p.R202H	1	Microphthalmia (1)
		c.717G>A	p.R239R		

Paznekas et al. 2009; Paznekas et al. 2003	No	c.605G>A	p.R202H	17	Microphthalmia (1), microcornea (2)

Nishat et al. 2012; Paznekas et al. 2009; Paznekas et al. 2003; and Amador et al. 2008	No	c.389T>C	p.I130T	17	Microphthalmia (4), hypotelorism (6), cataract (1), pale/atrophic optic disc (1), and short palpebral fissures (4)

Park et al. 2019; Hayashi et al. 2014; Paznekas et al. 2009; Debeer et al. 2005; and Paznekas et al. 2003	No	c.119C>T	p.A40V	17	Microphthalmia (9), hypertelorism (3), hypotelorism (4), short axial length (4), cataract (1), microcornea (8), thick cornea (4), macular hypoplasia (1), shallow anterior chamber (4), myopia (4), strabismus (6) (1 esotropic), glaucoma (6), and epicanthus (3)

**Table 6:** Mutant connexin-43 domains and associated phenotype.

*GJA1* mutation	Protein domain (amino acid range) (obtained from UniProt-P17302)	Associated phenotype (no. of individuals)
p.G2fsX7 (with p.R101X) p.G2V p.L11P p.L11F p.L11I p.L7V p.S5C	Cytoplasmic N-terminus(1-13)	Microcornea (7), microphthalmia (5), epicanthus (4), strabismus (3) (1 esotropic), short palpebral fissures (2), telecanthus (2), amblyopia (1), dysplastic fundus (1), optociliary vein (1), dysplastic optic disc (1), pale/atrophic optic disc (1), persistent pupillary membrane (1), myopia (3), hyperopia (1) (anisometropic), glaucoma (1), ptosis (1), entropion (1), madarosis (1), hypertelorism (1), and cataract (1)

p.W25C p. R33X p.I31M p.K23T p.G22E p.G21R p.S18P p.Y17S p.L26P	Transmembrane-1 (14-36)	Microcornea (21), microphthalmia (14), short palpebral fissures (11), persistent pupillary membrane (6), madarosis (6), epicanthus (6), glaucoma (5), anterior iris stroma hypoplasia (3), hypertelorism (2), cataract (2), iris abnormalities (2), blonde fundus (1), iridoschisis (1), deep anterior chamber (1), hyperopia (2), strabismus (7) (3 esotropic), amblyopia (1), nystagmus (1), ptosis (1), epiblepharon (1), nasolacrimal duct obstruction (1), and flared eyebrows (1) (medially flared)

p.Q57SfsTer6 p.R76H p.R76C p.R76S p.H74P p.S69Y p.P59H p.Q49dup p.F52dup p.Q49P p.Q49E p.Q49K p.E48K p.D47H p.E42Q p.V41_A44del p.V41L (with p.R127H (GJB2 mutation)) p.A40V p.P59S	Extracellular-1 (37-76)	Microphthalmia (32), microcornea (30), glaucoma (15) (2 closed-angle, 1 open-angle), hypertelorism (11), epicanthus (10), strabismus (9) (3 esotropic), short palpebral fissures (9), iris atrophy (peripupillary) (8), cataract (6), shallow anterior chamber (6), hypotelorism (5), short axial length (4), myopia (4), corneal farinata (4), telecanthus (3), iris abnormalities (2), eccentric pupils (2), persistent pupillary membrane (2), dysplastic fundus (1), dysplastic optic (1), macular hypoplasia (1), synechiae (1), ciliary body cysts (1), deep anterior chamber (1), hyperopia (1), ptosis (1), blepharophimosis (1), madarosis (1), nasolacrimal duct abnormalities (1), and low-voltage ERG (1)

p.Y98C p.V96A p.V96E p.V96M p.H95R p.L90V p.S86Y p.V96G	Transmembrane-2 (77-99)	Hypertelorism (5), microcornea (2), microphthalmia (3), glaucoma (3), strabismus (2) (1 esotropic), short palpebral fissures (2), eyelid mucosal hypertrophy (1), telecanthus (1), epicanthus (1), optic disc atrophy (1), hyperopia (1), myopia (1), strabismus (1), paracentral scotoma (1), madarosis (1), and delayed visual evoked potentials (1)

p.R101X (with p.G2fsX7) p.R101X p.T154N p.T154A p.R148Q p.R148Ter p.M147T p.G143S p.G138D p.G138R p.G138S p.K134N p.K134E p.I130T p.L113P p.E110D p.L106R p.L106P p.K102N p.I132_K133delinsM	Cytoplasmic-1 (100-154)	Microphthalmia (20), microcornea (18), short palpebral fissures (14), hypotelorism (14), glaucoma (9), myopia (7), epicanthus (5), cataract (3), strabismus (3), shallow anterior chamber (3), hypertelorism (2), opaque lens (1), optic disc hypervascularity (1), pale/atrophic optic disc (1), pale irides (1), iris abnormalities (2), astigmatism (1), Duane syndrome (1), ptosis (1), occipital subcortical white matter changes (1), and delayed visual evoked responses (1)

p.F169del	Transmembrane-3 (155-177)	Short palpebral fissures (1)

p.R202H (with p.R239R) p.R202H p.K206R p.S201F p.H194P	Extracellular-2 (178-208)	Microphthalmia (18), uveitis (8), glaucoma (8), microcornea (4), opaque cornea (2), thick choroid (2), cataract (1), shallow anterior chamber (1), nystagmus (2), and ptosis (1)

p.S220Y p.V216L	Transmembrane-4 (209-231)	Microphthalmia (1), glaucoma (1), microcornea (1), and persistent pupillary membrane (1)

p.Y230fsX236	Transmembrane-4 & cytoplasmic C-terminus (209-382)	Hypertelorism (2), hypotelorism (1), and flared eyebrows (2) (1 medially flared)

p.R239R (with p.R202H) p.I311P p.C260fsX306	Cytoplasmic C-terminus(232-382)	Short palpebral fissures (3), epicanthus (2), hypotelorism (2), microcornea (2), pale irides (2), myopia (2), hyperopia (1) (1 anisometropic), corneal opacity (1), microphthalmia (1), retinal dysplasia (1), choroid thinning (1), glaucoma (1), madarosis (1), and loss of flash ERG (1)

Missense mutation exon 2 (unspecified)	Unknown	Microphthalmia (1), cataract (1), microcornea (1), uveitis (1), glaucoma (1), epicanthus (1), telecanthus (1), short palpebral fissures (1), and ptosis (1)

## Supplementary Materials

Supplementary Materials Supplementary Material 1: all GJA1 mutations with associated eye and ocular adnexa features. This dataset groups patients with ODDD by GJA1 mutation and reports the associated eye and ocular adnexa features.
